# Aromatase inhibitors and antiepileptic drugs: a computational systems biology analysis

**DOI:** 10.1186/1477-7827-9-92

**Published:** 2011-06-21

**Authors:** Yagmur Muftuoglu, Gabriela Mustata

**Affiliations:** 1 Department of Biophysics and Chemical & Biomolecular Engineering, Johns Hopkins University, Baltimore, MD 21218, USA; 2 Department of Computational and Systems Biology, University of Pittsburgh, Pittsburgh, PA 15260, USA

## Abstract

**Background:**

The present study compares antiepileptic drugs and aromatase (CYP19) inhibitors for chemical and structural similarity. Human aromatase is well known as an important pharmacological target in anti-breast cancer therapy, but recent research demonstrates its role in epileptic seizures, as well. The current antiepileptic treatment methods cause severe side effects that endanger patient health and often preclude continued use. As a result, less toxic and more tolerable antiepileptic drugs (AEDs) are needed, especially since every individual responds differently to given treatment options.

**Methods:**

Through a pharmacophore search, this study shows that a model previously designed to search for new classes of aromatase inhibitors is able to identify antiepileptic drugs from the set of drugs approved by the Food and Drug Administration. Chemical and structural similarity analyses were performed using five potent AIs, and these studies returned a set of AEDs that the model identifies as hits.

**Results:**

The pharmacophore model returned 73% (19 out of 26) of the drugs used specifically to treat epilepsy and approximately 82% (51 out of 62) of the compounds with anticonvulsant properties. Therefore, this study supports the possibility of identifying AEDs with a pharmacophore model that had originally been designed to identify new classes of aromatase inhibitors. Potential candidates for anticonvulsant therapy identified in this manner are also reported. Additionally, the chemical and structural similarity between antiepileptic compounds and aromatase inhibitors is proved using similarity analyses.

**Conclusions:**

This study demonstrates that a pharmacophore search using a model based on aromatase inhibition and the enzyme's structural features can be used to screen for new candidates for antiepileptic therapy. In fact, potent aromatase inhibitors and current antiepileptic compounds display significant - over 70% - chemical and structural similarity, and the similarity analyses performed propose a number of antiepileptic compounds with high potential for aromatase inhibition.

## Background

The need to discover and develop new antiepileptic drugs (AEDs) is clear. The adverse side effects of the existent therapies - from cognitive impairment [1] to depression, anorexia, somnolence [2], and even birth defects [3] - have long been reported. Even the newer anticonvulsant medications have offered little relief [1,2]. In fact, harmful side effects seem to be the most significant factor in the approximately 35% long-term or 3-year retention rate for all new AEDs [1]. As a result, less toxic and more tolerable AEDs are needed, especially since every individual responds slightly differently to given treatment options. A greater number of less harmful AEDs could serve as helpful alternatives for those who do not respond well to current treatments.

One potential avenue for the design of more tolerable AEDs exists in the subset of small molecules that inhibit the enzyme aromatase (CYP19), a cytochrome P450 enzyme that catalyzes the conversion of androgens into estrogens. In fact, aminoglutethimide, a drug that had been originally pursued for anticonvulsant therapy, eventually became the first-generation aromatase inhibitor [4]. Interestingly, several antiepileptic drugs - like lamotrigine, oxcarbazepine (also known as oxacarbazepine), tiagabine, phenobarbital, phenytoin, ethosuximide, (ethosusuximide), and valproic acid (valproate) - are known to inhibit aromatase, with IC_50 _values ranging from 1.4 to 49.7 μM [5]. In fact, some aromatase inhibitors (AIs) - like letrozole, for example, which has been approved for the treatment of breast cancer by the Food and Drug Administration (FDA) [6] - have been clinically successful in treating epilepsy in men [7].

Aromatase inhibitors have been especially useful for treating male patients with sexual or reproductive dysfunction in addition to epilepsy [7]. They have also been prescribed clinically when the current antiepileptic treatment options lead to side effects severe enough to preclude continued use and even when AEDs proved ineffective in controlling epilepsy [8]. Sexual or reproductive abnormalities often seen in epileptic males are typically attributed to the fact that estrogens suppress sexual interest and function in males [9]. Estradiol in particular, the most potent estrogen in the human body [10], is found at elevated levels in men with epilepsy [11]. In fact, many clinical studies indicate that a statistically significant and relatively high percentage of individuals with epilepsy, whether male or female, also display sexual dysfunction [3,7-9,11], reproductive dysfunction [8,9,11], and hormonal or endocrine abnormalities [9,11] as would be expected from high estrogen levels.

Among women, the relationship between epilepsy and estrogen imbalance is especially striking. For example, it has been shown that around 56.5% of women with amenorrhea or anovulatory cycles have electroencephalography (EEG) abnormalities [9]. Seizure frequency can also be associated with perimenarche [12,13] menarche [9,12], menstruation [3,9,12-14], pregnancy [9,12], perimenopause [3,12,13], and menopause [3,9,12,13]. Catamenial epilepsy, a form of epilepsy in which the periodicity of seizure exacerbation corresponds closely with the menstrual cycle, is thought to affect from 30% to as much as 70% of epileptic females [3,9,12-14]. This type of epilepsy has been found to exist in three distinct patterns [12-14], and is thought to occur due to the surge of estrogen and withdrawal of progesterone at ovulation [3]. However, the periodicity of seizures is common in both genders [14].

Additionally, it has long been established that estrogens are epileptogenic [9] and have proconvulsive effects [12] in the human body. This is due to the ability of estrogens to lower the seizure threshold [9,14], to increase seizure discharges [9,14], and to increase neural membrane excitability [12-14]. However, there have also been some studies that hint at the potential anticonvulsant role of estrogens [13] and this highlights the complex role of sex hormones in the body and the complex etiology of epilepsy itself. The conclusiveness of such studies could indeed be clouded by other factors, especially since epileptic seizures in general are known to depend on many factors other than sex steroids.

For example, high progesterone levels, even during periods of relatively high estrogen levels, could still result in the antiepileptic effects witnessed in some patients, especially since seizure frequency correlates in a statistically significant manner with the serum estradiol:progesterone ratio [9]. In addition, it has been previously proposed that perhaps the different effects of estradiol and other estrogens on epileptic tissue actually depend on their final local concentrations [15]. The previous argument is further supported by the fact that estrogens could act as substrates for aromatase (a better terminology could be probably "false substrates"). Their reactions generate catechol estrogen, 2-hydroxyestrogen, and 6-hydroxyestrogen that may have critical roles in the induction or promotion of estrogen-responsive malignancies. Because of this, both androgens and estrogens act as competitive inhibitors of aromatase and derivatives of both androgens and estrogens have been tested as AIs [16].

All in all, however, the existence of a relationship between estrogen biosynthesis and epilepsy is unmistakable, and scientific evidence exists to prove that all forms of epilepsy may indeed have this common origin [17]. Regardless, the set of current antiepileptic drugs and the set of known aromatase inhibitors certainly have several, if not many, compounds in common. Similarly, screening for potential AIs could lead to the identification of potential AEDs, as well. The focus of this study, therefore, is to demonstrate this and to further analyze the common features among AIs and AEDs in an effort to prove their chemical and structural similarity.

## Methods

### Virtual screening

**Figure 1:**
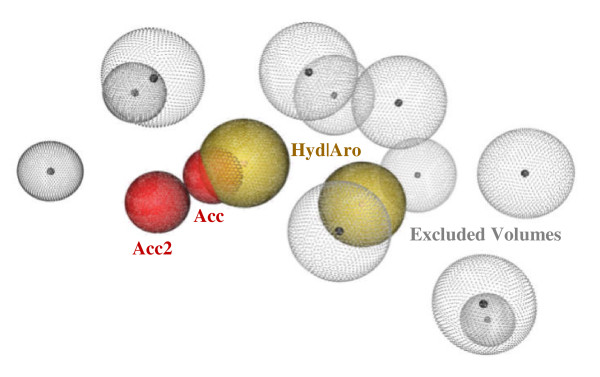
**Three-dimensional representation of the Merged pharmacophore model generated in previous work**[18]. Two hydrophobic groups (yellow spheres), two hydrogen-bond acceptors (red spheres), and 11 excluded volumes (gray spheres) are shown.

A previously generated pharmacophore model [18] is shown in Figure 1. To generate this model, a Training Set [18] of the 20 most potent, yet structurally-diverse aromatase inhibitors known and presented in the literature was created. These compounds were used to generate the ligand-based (LB) pharmacophore model on Molecular Operating Environment (MOE) [19]. The recently determined aromatase active site crystal structure (PDB code: E3QM) [20] was used to generate the structure-based (SB) pharmacophore model on LigandScout [21]. The SB model was based on the interactions that define aromatase inhibition, such as hydrophobic interactions, hydrogen bonding, and electrostatic interactions. The LB and SB models were merged to create the Merged Model with a root mean squared deviation (RMSD) value of 0.11 Å. All three models - the LB, SB, and Merged - were analyzed computationally, and the Merged Model was found superior to the original models by combining the strengths of both [18].

The Merged Model [18] was used to screen the set of FDA-approved drugs as provided by the DrugBank [22,23]. The "approved" set of DrugBank molecules was downloaded as an sdf file, and each compound was converted into an individual mol file with Molegro Virtual Docker (MVD) [24]. A database file was created using the Molecular Operating Environment (MOE) [19] by importing every mol file from the "approved" set of DrugBank molecules.

The compounds with molecular weight lower than 550 were screened with the Merged Model, which had been originally designed to screen for new classes of aromatase inhibitors [18]. This was accomplished by generating conformations for each "approved" drug and by and analyzing these for potential matches with the Merged Model through the conformation import function of the pharmacophore search function on MOE [19]. The hits related to epilepsy and seizure therapy were isolated by their DrugBank identification numbers.

Separately, conformations of every compound in the set of aromatase inhibitors previously used to create and to validate the pharmacophore model were generated through the conformation import feature of the pharmacophore search function on MOE [19]. For each compound, the conformation with the lowest root mean square deviation (RMSD) from the Merged pharmacophore model [18] was taken as the "ideal" conformation for that specific compound.

The "ideal" conformations - one for each of the AIs - were ranked according to RMSD. The five "ideal" conformations with the lowest RMSD values corresponded to five individual AIs, which will hereafter be referred to as the "top five AIs." Thus, the top five AIs were those capable of adopting a conformation with an RMSD value among the five lowest RMSD values from the Merged pharmacophore model. The top five AIs are shown in Additional file 1: **Table S1**.

The chemical fingerprints of the top five AIs and of the epilepsy-related hits were then calculated with the fingerprints Typed Graph Triangles (TGT) [25] and EigenSpectrum Shape (EShape3D) [26]. The TGT fingerprint encodes three-point pharmacophores from two-dimensional structures of the compounds. To each atom, it assigns a given molecule type - donor, acceptor, polar, anion, cation, or hydrophobe, - and all triplets are coded as features using three graph distances (bond count) and three atom types in each triplet. In contrast, ESshape3D aims at rapidly determining the shape similarity of molecules. The ESshape3D fingerprint starts by calculating a matrix with the Euclidean distances between all heavy atoms in molecule to thereafter form a spectrum characteristic of its shape with the matrix's eigenvalues. This spectrum is then encoded as a fingerprint, and the similarity score is calculated as the inverse of the distance between the corresponding fingerprints.

Therefore, the TGT and EShape3D fingerprint types were chosen based on the important characteristics - chemical features and shape of the molecule, respectively - that they represent because these are crucial for the especially substrate-specific binding pocket active site of the aromatase enzyme [18,19]. The similarity metric used with TGT was Tanimoto Coefficient; with EShape3D, it was Inverse Distance. The threshold at which top five AIs are found similar to the epilepsy-related hits was determined by successively adjusting the overlap value.

### Ingenuity Pathway Analysis (IPA)

Pathways analysis is a method that allows the visualization of biological pathways and that provides a greater understanding of diseases [27]. IPA consists of an extensive repository of biological and chemical knowledge, offering the researcher access to the most current findings available on genes, drugs, chemicals, protein families, normal cellular and disease processes, and signaling and metabolic pathways. The software allows one to search for information on genes and chemicals, their impact on diseases and cellular processes, and their role in signaling and metabolic pathways. One major problem that drug developers face is that many drugs share similar targets, as evidenced by phenotypic side-effects similarities [28], or that a given drug may be capable of targeting more than one protein. This is generally due to two effects: (1) target promiscuity (i.e. the promiscuity of the proteins themselves, their involvement in multiple functions, and their structural similarities, as well as their involvement in complex networks of interactions that simultaneously couple several cellular pathways), and (2) chemical similarities of many small molecule inhibitors as evidenced by the recent global mapping of the pharmacological space [29].

## Results

The AIs that were found to be in the top five were also experimentally found to be of high potency for inhibiting aromatase *in vitro *relative to other known AIs. The inhibitory half-maximal concentration (IC_50_) values of the top five AIs are shown in Additional file 1: **Table S1**. Interestingly, four out of five had been among the 20 most potent AIs that were used to generate the pharmacophore model. However, the final pharmacophore model with which the DrugBank database was screened for this study had been generated from both this set of 20 most potent AIs and from the active site crystal structure of the aromatase enzyme. Thus, these AIs represent those among the 20 most potent that closely match the chemical features and shape necessary for inhibition as dictated by the active site structure, as well, further demonstrating the essential role of the structure-based pharmacophore model [18].

The fifth AI - liarozole, - although inhibitory in the nanomolar range [30,31], had been deemed too similar in chemical structure to vorozole [30-32], one of the 20 most potent AIs in the Training Set used to generate the Merged Model; therefore, it was used to test the model rather than to generate it [18]. Regardless, it is identified as one of the AIs with the lowest RMSD from the pharmacophore model, as is vorozole. Vorozole is an AI currently being tested in Phase IV clinical trials [32], and anastrozole is already an FDA-approved drug [6] for treating breast cancer [4,6]. It is noted that aminoglutethimide, a first generation AI with severe toxic side effects that had originally been pursued as anticonvulsant therapy [4], was not found among the top five. Therefore, the top five AIs represent a set of compounds that are not biased toward any AEDs.

The Merged pharmacophore model [18] was found to identify many of the antiepileptic drugs contained in the FDA-approved subset of drugs in the DrugBank [22,23]. Among the 26 drugs in the FDA-Approved database of the DrugBank [22,23] specifically used to treat epilepsy, the Merged Model hit 19 (73%). Out of the 62 compounds listed as having anticonvulsant properties, a subset of which were the AEDs used for epilepsy therapy, the Merged Model returned 51 (82%). Additionally, the Merged Model [18] hit seven out of seven compounds that had been proven to have both anticonvulsant and aromatase-inhibiting affects [5] (100%). These compounds are shown in Additional file 2: **Table S2**. Results of the similarity analyses that were conducted on the entire set of AEDs with the top five AIs are shown in Additional file 3: **Table S3**.

**Figure 2:**
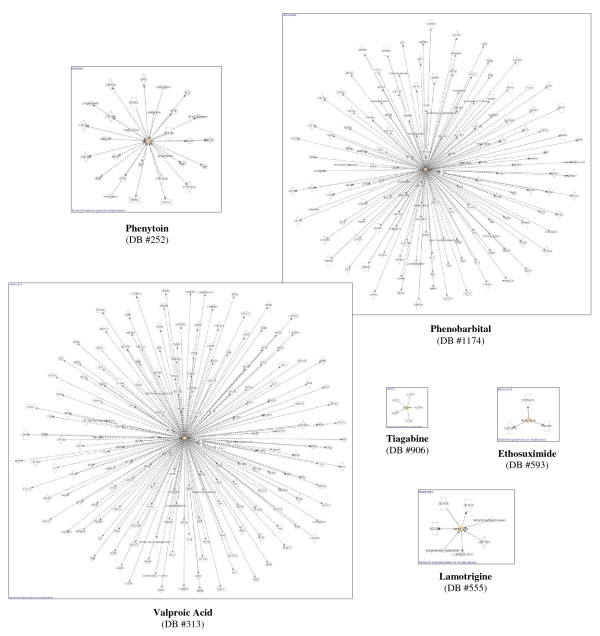
**Diagrams depicting interactions between six of the seven aromatase-inhibiting AEDs, oxcarbazepine (DB #776) not included, since information about its interactions was unavailable**. Specific molecule types are represented as: cytokines (square), growth factors (dotted square), small molecule drugs or toxicants (oval), enzymes (vertical diamond), G-protein coupled receptors (vertical rectangle), ion channels (dotted vertical rectangle), kinases (inverted traingle), ligand-dependent nuclear receptors (rectangle), peptidases (horizontal diamond), phosphatases (triangle), transcription regulators (horizontal ellipse), translation regulators (hexagon), transmembrane receptors (vertical ellipse), transporters (trapezoid), microRNA (inverted trapezoid), complexes (thick circle), and other important molecules (circle). Dotted lines indicate an indirect interaction, while solid lines indicate a direct interaction.

**Figure 3:**
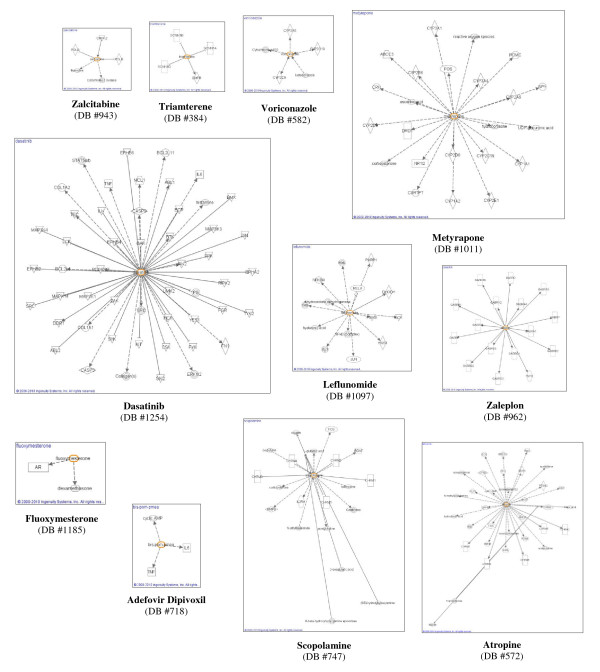
**Diagrams depicting multiple target interactions of the potential AEDs proposed by the Merged pharmacophore model search**. These compounds were chosen from among the set of molecules in Additional file 5: **Table S5 **for their structural diversity and oral-availability. Specific molecule types are represented as indicated in Figure 2 legend.

To understand the toxicity of the FDA approved AEDs, a pathway analysis with IPA was performed to identify the targets that are being affected by these molecules. The IPA analysis was applied to current AEDs as well as to the hits that resulted from the pharmacophore screening. As illustrated in Figures 2 and 3, with this program, targets are represented as geometrical shapes (i.e. circle, square, ellipse, etc.), and arrows connecting the molecule of interest to different targets represent a biological relationship that is supported by at least one published reference or by the IPA knowledge base. These maps can be multidimensional and provide information about cytokines, growth factors, small molecule drugs or toxicants, enzymes, G-protein coupled receptors, ion channels, kinases, ligand-dependent nuclear receptors, peptidases, phosphatases, transcription regulators, translation regulators, transmembrane regulators, transporters, microRNA, complexes, and other important molecules.

## Discussion

A key result of this study is the identification of AEDs that the similarity search methods used - TGT and EShape3D - identify to be similar to a given top five AI by at least an overlap value of 70%. Additional file 3: **Table S3 **shows the results of these similarity analyses, in which the set of antiepileptic drugs were analyzed for similarity with the top five AIs according to fingerprint methods TGT and EShape3D. If an AED was identified to be similar with a top five AI by at least one of the fingerprint methods, its DrugBank identification number was written in Additional file 3: **Table S3 **corresponding to the top five AI with which it was found similar and to the overlap values - 70% or higher - at which it was found similar.

Among these compounds, those that were found similar to one of the top five AIs at an overlap value of at least 70% by both fingerprint methods were reported in bolded text in Additional file 3: **Table S3**, with the two-dimensional structures shown in Additional file 4: **Table S4**. These compounds are, therefore, considered to have high probability to inhibit the human aromatase enzyme because they have been identified as similar to a top five AI in terms of chemical features and also according to compound shape. In this manner, the similarity search analysis very clearly proposes five AEDs with potential for aromatase inhibition. These five AEDs have not been assayed for aromatase inhibition according to the literature. Several AEDs that actually do inhibit aromatase - lamotrigine (DB #555), oxcarbazepine (DB #776), tiagabine (#906), phenobarbital (DB #1174), and phenytoin (DB #252) - were identified through this method, whether by the TGT or by the Eshape3D fingerprint type. These compounds, as well as the five proposed by the similarity search method, are shown in Additional file 4: **Table S4 **to further highlight their structural similarity.

In fact, the only aromatase-inhibiting AEDs not picked up through the similarity analyses - but still hit by the Merged Model - are ethosuximide (DB #593) and valproic acid (DB #313). Valproic acid, with a structure rather unlike other aromatase-inhibiting AEDs, is likely to bind differently to the aromatase active site. As a result, it may be considered as irrelevant to the similarity analyses. Ethosuximide, on the other hand, does closely resemble the chemical structures of some of the other aromatase-inhibiting AEDs identified through the similarity analyses but was not similar enough to the top five AIs to be identified at 70% overlap.

Regardless, the similarity search method did, in general, hit several compounds that are both AIs as well as AEDs. More importantly, however, these analyses speak to the relative similarity between potent AIs and current antiepileptic drugs. In addition, it has become apparent that a pharmacophore search with a model - originally designed to find new classes of AIs - could also yield potential new AEDs that are less toxic and more tolerable. The pharmacophore search of the FDA-approved set of compounds from the DrugBank offers some potential leads for accomplishing this. In Additional file 5: **Table S5 **are reported the compounds that were hit by the Merged Model with a very low RMSD value, less than or equal to 0.20 Å. As shown here, already FDA-approved treatments offer potential for inhibiting aromatase. It is noted that, in fact, Lamotrigine (DB #555), a current antiepileptic drug that was also proposed for aromatase inhibition through the similarity analyses, falls in the set of Merged Model hits with RMSD less than or equal to 0.20 Å, as well.

Interactions between the seven aromatase-inhibiting AEDs in Additional file 2: **Table S2 **and multiple targets were obtained through the IPA analysis [27]. Diagrams depicting these interactions [27] are shown in Figure 2 for six of the seven aromatase inhibiting AEDs (information about the interactions of oxcarbazepine (DB #776) was unavailable). Interactions [27] of the structurally-diverse and orally-available compounds among the proposed AEDs from Additional file 5: **Table S5 **were also obtained, and these are depicted below in Figure 3. These compounds were chosen specifically for their diverse structural characteristics and their oral availability as therapeutic agents.

Additionally, the interaction information was compiled into Additional file 6 &7: **Table S6 and S7 **so that a breakdown of these interactions for each compound could be obtained. For example, Additional file 6: **Table S6 **shows the number of specific types of proteins - such as cytokines, enzymes, kinases, etc. - on which each aromatase-inhibiting AED has an influence. Additional file 7: **Table S7 **shows the same for the proposed AEDs from Additional file 5: **Table S5**. In general, the compounds with relatively fewer off-target effects offer the most promise for developing less toxic, more tolerable AEDs; therefore, it can be seen that some of the compounds in the set of FDA-approved drugs that were hit by the Merged pharmacophore model with an RMSD value less than or equal to 0.20 Å do, indeed, show some potential.

## Conclusions

All in all, this study has shown that a pharmacophore model [18] - originally derived from potent AIs and from the aromatase active site structure - can be used to screen for AEDs. The screening of the FDA-approved subset of compounds from the DrugBank [22,23] with the Merged Model [18] returned 45 compounds with RMSD less than or equal to 0.20 Å that offer promise as potential AEDs. Through this study, the similarity between AIs and AEDs, with regard to both structure and chemical features, has also been shown. In fact, through these analyses, several AIs with significant similarity (over 70%) with some of the top five AIs were identified as having potential for aromatase-inhibition, as well.

The most important conclusions from this study are that: (1) a very good percentage of AEDs (73%) and of anticonvulsant compounds (82%) fit the pharmacophore model originally generated to screen for different classes of AIs, and (2) since several AEDs are also very similar to several AIs - specifically, the top five - this pharmacophore model can be used to find less toxic, more tolerable AEDs.

Additionally, it can be argued that the chemical and structural similarity of these two sets of compounds hints at the potentially significant role of aromatase in epilepsy. These findings offer leads into prospective new strategies for epilepsy treatment, made especially valuable given the challenges involved in treating this condition. For example, it is known that 30% of the newly diagnosed patients do not respond sufficiently to monotherapy [5]. Therefore, compounds capable of inhibiting aromatase should be investigated for potential in polytherapy, based on mechanism of action, in addition to being tested for antiepileptic properties. Specifically, with regard to men with epilepsy, current clinical data suggests that aromatase inhibitors could possibly enhance patient responses to antiepileptic treatments and that compounds capable of inhibiting aromatase should be analyzed for potential use in antiepileptic treatments [7]. Additionally, toxicity issues and adverse side effects incurred with the current treatment options for epilepsy can be avoided through the design of new, less harmful drugs for antiepileptic therapy. It has been clinically determined through recent studies that, at least in the short term, men who are prescribed aromatase inhibitors for conditions that stem from hormonal imbalances do not show signs of bone thinning or loss of bone density [10]. In that sense, inhibitors of the human aromatase enzyme seem to have great potential for use in antiepileptic treatments.

As a form of validation for the similarity analyses, AEDs proposed for potential aromatase inhibition, as shown in Additional file 4: **Table S4**, should be assayed *in vivo*. The potential AEDs shown in Additional file 5: **Table S5 **- with the exception of Lamotrigine (DB #555) - are clear examples of compounds that could be assayed both for aromatase inhibition and for anticonvulsant properties, as well. In addition, the Merged Model generated in previous work [18] can be used to screen databases other than the FDA-approved set of compounds analyzed in this study. In this way, less toxic and more tolerable AEDs could be identified for the treatment of epilepsy.

## Competing interests

The authors declare that they have no competing interests.

## Authors' contributions

YM performed the screening and similarity analyses and drafted the manuscript. GM conceived the study and helped draft the manuscript. Both authors read and approved the final manuscript.

## Supplementary Material

Additional file 1 **Top five aromatase inhibitors**. Top five AIs identified as having lowest RMSD values from the Merged pharmacophore model generated and explained in previous work [18][file cites [4,6,30-34]]. Click here for file

Additional file 2 **Aromatase-inhibiting AEDs hit by the Merged Model**. Seven out of seven aromatase-inhibiting antiepileptic drugs [5] hit by the Merged pharmacophore model [18]. Click here for file

Additional file 3 **Results of similarity analyses AEDs hit by the Merged Model**. Results of similarity analyses performed on the set of AEDs hit by the Merged pharmacophore model [18]. Compounds that were found similar to one of the top five AIs at an overlap value of at least 70% by both fingerprint methods are reported in bolded text. Click here for file

Additional file 4 **Aromatase-inhibiting AEDs identified and AEDs proposed to be aromatase-inhibiting by similarity analyses**. Aromatase-inhibiting antiepileptic drugs identified by similarity analyses and AEDs proposed to be aromatase-inhibiting through similarity analyses. Click here for file

Additional file 5 **FDA-approved Hits with RMSD less than or equal to 0.20 Å**. FDA-approved Merged pharmacophore model [18] hits with RMSD less than or equal to 0.20 Å Click here for file

Additional file 6 **Breakdown of the protein interactions of the aromatase-inhibiting AEDs**. Protein interactions of the aromatase-inhibiting AEDs broken down into specific types of proteins as indicated in Figure 2 legend. Click here for file

Additional file 7 **Breakdown of the protein interactions of the proposed AEDs**. Protein interactions of the proposed AEDs from Additional file 5: **Table S5 **broken down into specific types of proteins as indicated in Figure 2 legend. Click here for file
