# Supplementary material for: Aromatase inhibitors and antiepileptic drugs: a computational systems biology analysis
Source: Reprod Biol Endocrinol. 2011 Jun 21;9:92. doi: 10.1186/1477-7827-9-92 (PMC3129585; doi:10.1186/1477-7827-9-92)
Supplement: Additional file 1 — Top five aromatase inhibitors. Top five AIs identified as having lowest RMSD values from the Merged pharmacophore model generated and explained in previous work [18][file cites [4,6,30-34]]. [file 1477-7827-9-92-S1.DOC]

| **Name** | **Structure** | **RMSD** | **IC50 (nM)** |
| --- | --- | --- | --- |
| **1-(4-Fluorobenzyl)-3-(1H-1–imidazolylmethy1)-IH-indole** [33] | 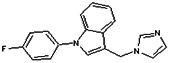 | 0.19 | 71.8 [33] |
| **5-[(Imidazol-1-yl)methyl]-5,6,7,8-tetrahydroquinoline** [34] | 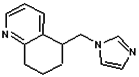 | 0.21 | 160 [34] |
| **Anastrozole** [4,6] | 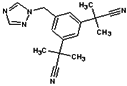 | 0.24 | 15 [4] |
| **Vorozole** [30,31,32] | 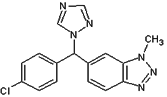 | 0.29 | 1.4 [30,31] |
| **Liarozole** [30,31] | 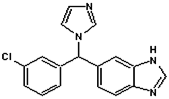 | 0.29 | 4 [30,31] |
