# Supplementary material for: Aromatase inhibitors and antiepileptic drugs: a computational systems biology analysis
Source: Reprod Biol Endocrinol. 2011 Jun 21;9:92. doi: 10.1186/1477-7827-9-92 (PMC3129585; doi:10.1186/1477-7827-9-92)
Supplement: Additional file 2 — Aromatase-inhibiting AEDs hit by the Merged Model. Seven out of seven aromatase-inhibiting antiepileptic drugs [5] hit by the Merged pharmacophore model [18]. [file 1477-7827-9-92-S2.DOC]

| **Compound Name (DB#)** [22,23] | **Structure**  **(RMSD from Model** [18]**)** | **Delivery Method(s)** [22,23] | **Additional Interactions** [27] |
| --- | --- | --- | --- |
| **Phenytoin**  (DB #252) | 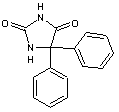  (0.56 Å) | oral  intravenous  intramuscular | **Inhibits**: Sodium Channel mammalia complex(es), SCN1B and SCN1A human protein  **Molecular Functions**: not available  **Biological Processes**: not available |
| **Valproic Acid**  (DB #313) | 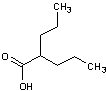  (0.65 Å) | oral  intravenous | **Inhibits**: Hdac, HDAC1, HDAC5, HDAC2, HDAC7, HDAC3, HDAC4, GSK3B, HDAC8, HDAC10, HDAC9, HDAC6 mammalian proteins CYP2C9, CYP2A6, GAD2, HDAC1, ABAT, Hdac, ALDH5A1, CYP3A4, HIF1A, CYP2C19, F3 human proteins, Akr1b1 rat protein, Histone deacetylase mammalia and human complex(es), Hdac mammalian, Gpd mammalian  **Molecular Functions**: not available  **Biological Processes**: not available |
| **Lamotrigine**  (DB #555) | 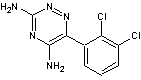  (0.14 Å) | oral | **Inhibits**: Sodium Channel mammalia complex(es), Scn2b rat protein, Scn2a1 rat protein, Glutamate Receptor mammalia complex(es), SCN1A human protein  **Molecular Functions**: antiepileptic drug, chemical drug, inhibitor  **Biological Processes**: apoptosis of neurons, flow of blood, swimming behavior of mice, locomotion of mice, release of L-glutamic acid |
| **Ethosuximide**  (DB #593) | 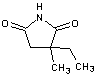  (0.78 Å) | oral | **Inhibits**: CACNA1H, CACNA1G, CACNA1I human proteins  **Molecular Functions**: antiepileptic drug, chemical drug  **Biological Processes**: not available |
| **Oxcarbazepine**  (DB #776) | 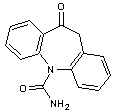  (0.95 Å) | oral | **Inhibits**: not available  **Molecular Functions**: antiepileptic drug, chemical drug, receptor antagonist  **Biological Processes**: not available |
| **Tiagabine**  (DB #906) | 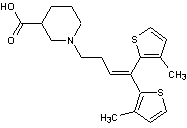  (0.66 Å) | oral | **Inhibits**: SLC6A12, SLC32A1, SLC6A13, SLC6A1, SLC6A11 human proteins, Slc6a1 rat protein  **Molecular Functions**: antiepileptic drug, chemical drug, inhibitor  **Biological Processes**: antinociception of mice, bodily balance of mice |
| **Phenobarbital**  (DB #1174) | 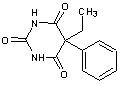  (0.52 Å) | oral  intramuscular | **Inhibits**: TCF20 mammalia, CYP3A41 mammalia  **Molecular Functions**: not available  **Biological Processes**: not available |
