# Supplementary material for: Aromatase inhibitors and antiepileptic drugs: a computational systems biology analysis
Source: Reprod Biol Endocrinol. 2011 Jun 21;9:92. doi: 10.1186/1477-7827-9-92 (PMC3129585; doi:10.1186/1477-7827-9-92)
Supplement: Additional file 3 — Results of similarity analyses AEDs hit by the Merged Model. Results of similarity analyses performed on the set of AEDs hit by the Merged pharmacophore model [18]. Compounds that were found similar to one of the top five AIs at an overlap value of at least 70% by both fingerprint methods are reported in bolded text. [file 1477-7827-9-92-S3.DOC]

| **Top Five AI** | **Fingerprint** | **Metric** | **Overlap between AED and Top Five AI** | **DB#s of similar AEDs** |
| --- | --- | --- | --- | --- |
| **1-(4-Fluoro-benzy1)-3-1(H-I–imidazolyl-methy1)-IH-indole** [33,34]  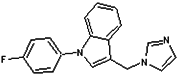 | TGT | Tanimoto Coefficient | 85% |  |
|  |  |  | 80% |  |
|  |  |  | 75% | 349, 425, 5246 |
|  |  |  | 70% | **349, 425,** 5246, 832 |
|  | ESshape3D | Inverse Distance | 85% | 1595 |
|  |  |  | 80% | 349, 1595, 628, 1068 |
|  |  |  | 75% | 349, 425, 1595, 628, 1068, 837 |
|  |  |  | 70% | **349**, **425**, 252, 776, 1595, 628, 1068, 837 |
| **5-[(Imidazol-1-yl)-methyl]-5,6,7,8-tetrahydroquinoline** [34]  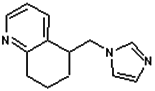 | TGT | Tanimoto Coefficient | 85% | 425 |
|  |  |  | 80% | 5246, 425, 349, |
|  |  |  | 75% | 5246, 832, 425, 349, |
|  |  |  | 70% | **5246**, **832**, **311**, 349, 617, 425, 776, |
|  | ESshape3D | Inverse Distance | 85% | 555, 532, 750, 311 |
|  |  |  | 80% | 555, 5246, 532, 754, 750, 311, 1174 |
|  |  |  | 75% | 555, 5246, 532, 754, 750, 311, 1174 |
|  |  |  | 70% | **5246**, **832**, **311**, 463, 532, 754, 1056, 750, 909, 1174, 849, 564, 961, 555 |
| **Anastrozole** [4,6]  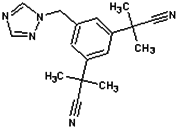 | TGT | Tanimoto Coefficient | 85% |  |
|  |  |  | 80% | 425 |
|  |  |  | 75% | 349, 425, 5246 |
|  |  |  | 70% | **349, 425**, 5246, 832 |
|  | ESshape3D | Inverse Distance | 85% | 628, 1068, 425, 837 |
|  |  |  | 80% | 349, 425, 1595, 628, 1068, 837 |
|  |  |  | 75% | 349, 425, 1595, 628, 1068, 837 |
|  |  |  | 70% | **349, 425**, 1595, 628, 1068, 837 |
| **Vorozole** [30,31,32]  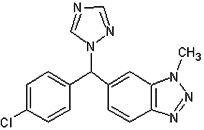 | TGT | Tanimoto Coefficient | 85% |  |
|  |  |  | 80% | 425 |
|  |  |  | 75% | 425 |
|  |  |  | 70% | **349, 425**, 5246, 311 |
|  | ESshape3D | Inverse Distance | 85% | 425, 837 |
|  |  |  | 80% | 628, 1068, 425, 837 |
|  |  |  | 75% | 628, 1068, 425, 837, 1320 |
|  |  |  | 70% | **349, 425**, 1595, 628, 1068, 837, 1320, 906 |
| **Liarozole** [30,31]  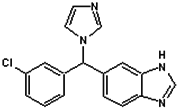 | TGT | Tanimoto Coefficient | 85% |  |
|  |  |  | 80% | 425 |
|  |  |  | 75% | 425 |
|  |  |  | 70% | **349, 425**, 5246, 311 |
|  | ESshape3D | Inverse Distance | 85% | 425, 837 |
|  |  |  | 80% | 628, 1068, 425, 837 |
|  |  |  | 75% | 628, 1068, 425, 837, 1320 |
|  |  |  | 70% | **349, 425**, 1595, 628, 1068, 837, 1320, 906 |
