# Supplementary material for: Aromatase inhibitors and antiepileptic drugs: a computational systems biology analysis
Source: Reprod Biol Endocrinol. 2011 Jun 21;9:92. doi: 10.1186/1477-7827-9-92 (PMC3129585; doi:10.1186/1477-7827-9-92)
Supplement: Additional file 4 — Aromatase-inhibiting AEDs identified and AEDs proposed to be aromatase-inhibiting by similarity analyses. Aromatase-inhibiting antiepileptic drugs identified by similarity analyses and AEDs proposed to be aromatase-inhibiting through similarity analyses. [file 1477-7827-9-92-S4.DOC]

| **Structure, Name (DrugBank#)** [22,23]**, and RMSD from Merged Model** [18] | | |
| --- | --- | --- |
| **Aromatase-inhibiting AEDs identified by similarity** | | **AEDs proposed by**  **similarity method** |
| 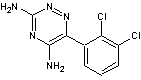  **Lamotrigine** (DB #555) 0.14 Å | 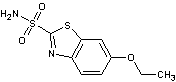  **Ethoxzolamide** (DB #311) 0.32 Å | |
| 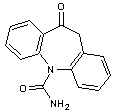  **Oxcarbazepine** (DB #776) 0.95 Å | 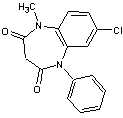  **Clobazam** (DB #349) 0.40 Å | |
| 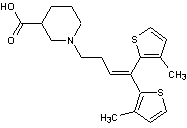  **Tiagabine** (DB #906) 0.66 Å | 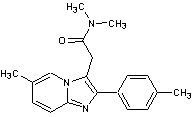  **Zolpidem** (DB #425) 0.32 Å | |
| 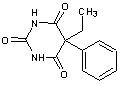  **Phenobarbital** (DB #1174) 0.52 Å | 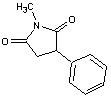  **Phensuximide** (DB #832) 0.62 Å | |
| 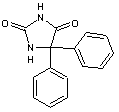  **Phenytoin** (DB #252) 0.56 Å | 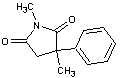  **Methsuximide** (DB #5246) 0.56 Å | |
