# Supplementary material for: Aromatase inhibitors and antiepileptic drugs: a computational systems biology analysis
Source: Reprod Biol Endocrinol. 2011 Jun 21;9:92. doi: 10.1186/1477-7827-9-92 (PMC3129585; doi:10.1186/1477-7827-9-92)
Supplement: Additional file 5 — FDA-approved Hits with RMSD less than or equal to 0.20 Å. FDA-approved Merged pharmacophore model [18] hits with RMSD less than or equal to 0.20 Å [file 1477-7827-9-92-S5.DOC]

| **Name (DrugBank#)** | **Structure** | **RMSD (Å)** |  | **Name (DrugBank#)** | **Structure** | **RMSD (Å)** |  | **Name (DrugBank#)** | **Structure** | **RMSD (Å)** |
| --- | --- | --- | --- | --- | --- | --- | --- | --- | --- | --- |
| **Decitabine**  (DB #1262) | 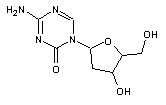 | 0.08 |  | **Cefmetazole**  (DB #274) | 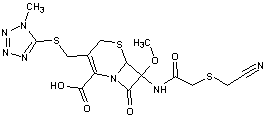 | 0.16 |  | **Atropine**  (DB #572) | 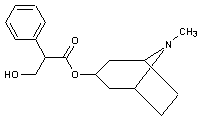 | 0.18 |
| **Azacitidine**  **(DB #**928) | 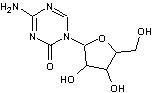 | 0.08 |  | **Alfentanil** (DB #802) | 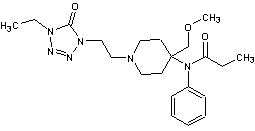 | 0.16 |  | **Grepafloxacin**  (DB #365) | 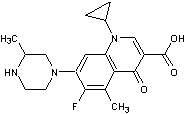 | 0.18 |
| **Cytarabine**  (DB #987) | 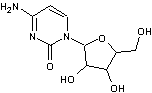 | 0.08 |  | **Dasatinib**  (DB #1254) | 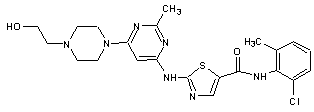 | 0.16 |  | **Adefovir Dipivoxil**  (DB #718) | 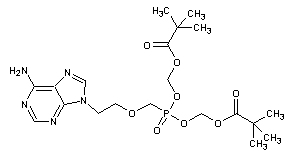 | 0.19 |
| **Lamivudine**  **(DB #**709) | 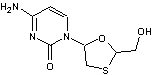 | 0.08 |  | **Leflunomide**  (DB #1097) | 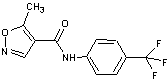 | 0.16 |  | **Dipyridamole**  (DB #975) | 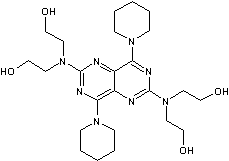 | 0.19 |
| **Emtricitabine** (DB #879) | 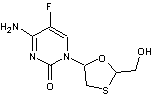 | 0.08 |  | **Nelarabine**  (DB #1280) | 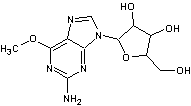 | 0.16 |  | **Ipratropium**  (DB #332) | 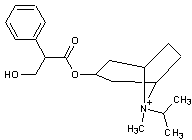 | 0.19 |
| **Zalcitabine**  (DB #943) | 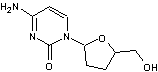 | 0.08 |  | **Didanosine**  (DB #900) | 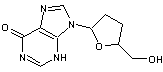 | 0.16 |  | **Quinine**  (DB #468) | 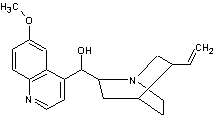 | 0.19 |
| **Triamterene**  (DB #384) | 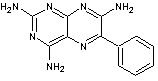 | 0.08 |  | **Fludarabine**  (DB #1073) | 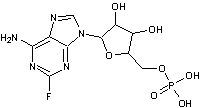 | 0.16 |  | **Rosoxacin**  (DB # 817) | 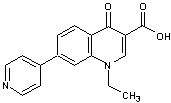 | 0.19 |
| **Phenazo-pyridine**  (DB #1438) | 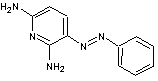 | 0.10 |  | **Cladribine**  (DB #242) | 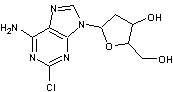 | 0.16 |  | **Methylscopol-amine**  (DB #462) | 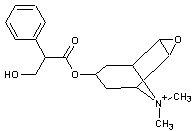 | 0.19 |
| **Metyrapone**  **(DB #**1011) | 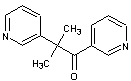 | 0.14 |  | **Clofarabine**  (DB #631) | 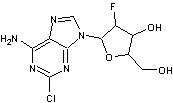 | 0.16 |  | **Irbesartan**  **(DB #**1029) | 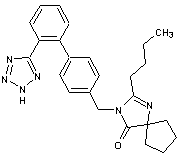 | 0.20 |
| **Cidofovir**  (DB #369) | 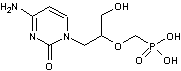 | 0.14 |  | **Alizapride**  (DB #1425) | 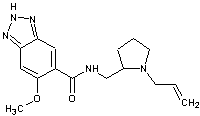 | 0.16 |  | **Forasartan**  (DB #1342) | 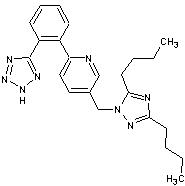 | 0.20 |
| **Lamotrigine**  (DB #555) | 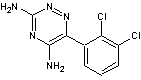 | 0.14 |  | **Dapiprazole**  (DB #298) | 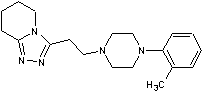 | 0.16 |  | **Valsartan**  (DB #177) | 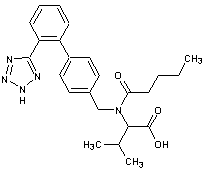 | 0.20 |
| **Voriconazole**  (DB #582) | 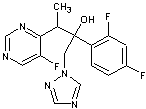 | 0.14 |  | **Tinidazole**  **(DB #**911) | 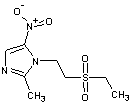 | 0.17 |  | **Losartan**  (DB #678) | 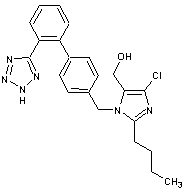 | 0.20 |
| **Sulfadoxine**  (DB #1299) | 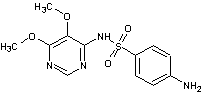 | 0.14 |  | **Zaleplon**  **(DB #**962) | 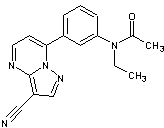 | 0.17 |  | **Candesartan**  (DB #796) | 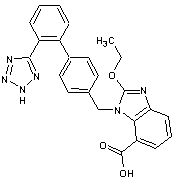 | 0.20 |
| **Tiludronate**  (DB #1133) | 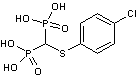 | 0.14 |  | **Fluoxy-mesterone**  **(DB #**1185) | 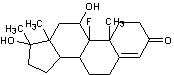 | 0.17 |  | **Tasosartan**  (DB #1349) | 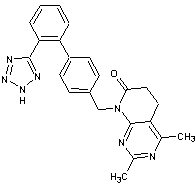 | 0.20 |
| **Cinoxacin**  (DB #827) | 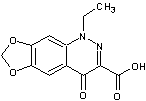 | 0.15 |  | **Scopolamine**  **(DB #**747) | 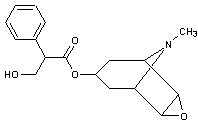 | 0.18 |  | **Celecoxib**  **(DB #**482) | 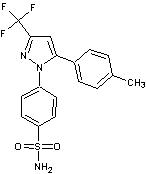 | 0.20 |
