# Supplementary material for: Aromatase inhibitors and antiepileptic drugs: a computational systems biology analysis
Source: Reprod Biol Endocrinol. 2011 Jun 21;9:92. doi: 10.1186/1477-7827-9-92 (PMC3129585; doi:10.1186/1477-7827-9-92)
Supplement: Additional file 6 — Breakdown of the protein interactions of the aromatase-inhibiting AEDs. Protein interactions of the aromatase-inhibiting AEDs broken down into specific types of proteins as indicated in Figure 2 legend. [file 1477-7827-9-92-S6.DOC]

| **IPA Symbol for Molecule** [27] | **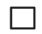** | **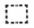** | **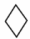** | **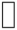** | **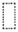** | **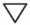** | **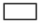** | **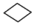** | **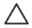** | **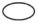** | **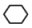** | **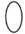** | **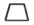** | **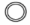** | **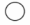** |
| --- | --- | --- | --- | --- | --- | --- | --- | --- | --- | --- | --- | --- | --- | --- | --- |
| **Type of Molecule -----------------------------------**  **Name of aromatase-inhibiting AED (DB#)** | **cytokines** | **growth factors** | **enzymes** | **G-protein coupled receptors** | **ion channels** | **kinases** | **ligand-dependant nuclear receptors** | **peptidases** | **phosphatases** | **transcription regulators** | **translation regulators** | **transmembrane receptors** | **transporters** | **complexes** | **other important molecules** |
| **Phenytoin** (DB #252) | **2** |  | **1** |  | **2** | **4** |  |  |  | **1** |  |  |  |  | **1** |
| **Valproic Acid** (DB #313) | **2** | **1** | **13** | **5** | **1** | **6** | **5** |  |  | **11** |  | **4** |  |  | **1** |
| **Lamotrigine** (DB #555) |  |  |  |  | **3** |  |  |  |  |  |  |  |  |  |  |
| **Ethosuximide** (DB #593) |  |  |  |  | **3** |  |  |  |  |  |  |  |  |  |  |
| **Oxcarbazepine** (DB #776) | **no information available** | | | | | | | | | | | | | | |
| **Tiagabine** (DB #906) |  |  |  |  |  |  |  |  |  |  |  |  | **5** |  |  |
| **Phenobarbital** (DB #1174) |  |  | **3** | **1** | **15** | **6** |  |  |  | **1** |  |  |  |  | **9** |
