# Supplementary material for: Aromatase inhibitors and antiepileptic drugs: a computational systems biology analysis
Source: Reprod Biol Endocrinol. 2011 Jun 21;9:92. doi: 10.1186/1477-7827-9-92 (PMC3129585; doi:10.1186/1477-7827-9-92)
Supplement: Additional file 7 — Breakdown of the protein interactions of the proposed AEDs. Protein interactions of the proposed AEDs from Additional file 5: Table S5 broken down into specific types of proteins as indicated in Figure 2 legend. [file 1477-7827-9-92-S7.DOC]

| **IPA Symbol for Molecule** [27] | **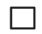** | **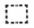** | **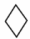** | **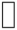** | | **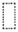** | **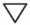** | **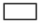** | **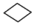** | **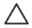** | **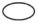** | **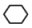** | **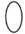** | **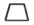** | **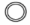** | **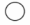** |
| --- | --- | --- | --- | --- | --- | --- | --- | --- | --- | --- | --- | --- | --- | --- | --- | --- |
| **Type of Molecule** ------------------------------------ **Name of experimental compound (DB#)** | **cytokines** | **growth factors** | **enzymes** | **G-protein coupled receptors** | | **ion channels** | **kinases** | **ligand-dependant nuclear receptors** | **peptidases** | **phosphatases** | **transcription regulators** | **translation regulators** | **transmembrane receptors** | **transporters** | **complexes** | **other important molecules** |
| **Decitabine** (DB #1262) | **1** | **1** | **16** | **7** | | **2** | **4** | **5** | **3** |  | **1** |  | **2** | **1** |  | **13** |
| **Azacitidine** (DB #928) |  |  | **6** |  | | **1** | **2** | **4** |  |  | **1** |  | **1** | **1** |  | **3** |
| **Cytarabine** (DB #987) | **1** |  | **7** |  | |  | **9** | **2** | **1** |  | **1** |  | **1** |  |  | **1** |
| **Lamivudine** (DB #709) | no information available | | | | | | | | | | | | | | | |
| **Emtricitabine** (DB #879) | no information available | | | | | | | | | | | | | | | |
| **Zalcitabine** (DB #943) |  |  | **2** | |  |  |  |  |  |  |  |  |  |  |  |  |
| **Triamterene** (DB #384 |  |  | **1** | |  | **3** |  |  |  |  |  |  |  |  |  |  |
| **Phenazopyridine** (DB #1438) | no information available | | | | | | | | | | | | | | | |
| **Metyrapone** (DB #1011) |  |  | **1** | | **1** |  |  |  |  |  |  |  |  |  |  |  |
| **Cidofovir** (DB #369) |  |  | **3** | |  |  |  |  |  |  |  |  |  |  |  |  |
| **Lamotrigine** (DB #555) |  |  |  | |  | **3** |  |  |  |  |  |  |  |  |  |  |
| **Voriconazole** (DB #582) | no information available | | | | | | | | | | | | | | | |
| **Sulfadoxine** (DB #1299) | no information available | | | | | | | | | | | | | | | |
| **Tiludronate** (DB #1133) | no information available | | | | | | | | | | | | | | | |
| **Cinoxacin** (DB #827) | no information available | | | | | | | | | | | | | | | |
| **Cefmetazole** (DB #274) | no information available | | | | | | | | | | | | | | | |
| **Alfentanil** (DB #802) |  |  |  | | **4** |  |  |  |  |  |  |  |  |  |  |  |
| **Dasatinib** (DB #1254) | **2** |  |  | |  |  | **11** |  | **1** |  |  |  |  |  |  | **8** |
| **Leflunomide** (DB #1097) |  |  | **2** | |  |  | **3** |  |  |  | **1** |  |  |  |  |  |
| **Nelarabine** (DB #1280) |  |  | **4** | |  |  |  |  |  |  |  |  |  |  |  |  |
| **Didanosine** (DB #900) | no information available | | | | | | | | | | | | | | | |
| **Fludarabine** (DB #1073) |  | **1** | **1** | |  |  | **8** |  | **1** |  |  |  | **2** |  |  |  |
| **Cladribine** (DB #242) | **1** |  |  | |  |  | **1** |  | **1** |  |  |  |  |  |  |  |
| **Clofarabine** (DB #631) |  |  | **5** | | **3** |  |  |  |  |  |  |  |  |  |  |  |
| **Alizapride** (DB #1425) | no information available | | | | | | | | | | | | | | | |
| **Dapiprazole** (DB #298) |  |  |  | | **6** |  |  |  |  |  |  |  |  |  |  |  |
| **Tinidazole** (DB #911) | no information available | | | | | | | | | | | | | | | |
| **Zaleplon** (DB #962) |  |  |  | |  | **15** |  |  |  |  |  |  | **1** |  |  |  |
| **Fluoxymesterone** (DB #1185) |  |  |  | |  |  |  | **1** |  |  |  |  |  |  |  |  |
| **Scopolamine** (DB #747) |  |  |  | | **5** |  |  |  |  |  |  |  | **1** |  |  |  |
| **Atropine** (DB #572) |  |  | **4** | | **5** |  |  |  |  |  |  |  |  |  |  |  |
| **Grepafloxacin** (DB #365) | **4** |  |  | |  | **1** | **4** |  |  |  |  |  |  |  |  |  |
| **Adefovir Dipivoxil** (DB #718) | **2** |  |  | |  |  |  |  |  |  |  |  |  |  |  |  |
| **Dipyridamole** (DB #975) | **1** |  | **11** | | **1** |  |  |  |  |  |  |  |  |  |  | **2** |
| **Ipratropium** (DB #332) |  |  |  | | **5** |  |  |  |  |  |  |  |  |  |  |  |
| **Quinine** (DB #468) |  |  | **1** | |  |  |  |  |  |  |  |  |  |  |  |  |
| **Rosoxacin** (DB # 817) | no information available | | | | | | | | | | | | | | | |
| **Methylscopolamine** (DB #462) |  |  |  | | **5** |  |  |  |  |  |  |  |  |  |  |  |
| **Irbesartan** (DB #1029) |  |  | **2** | | **2** |  | **5** |  |  |  |  |  | **1** |  |  | **2** |
| **Forasartan** (DB #1342) | no information available | | | | | | | | | | | | | | | |
| **Valsartan** (DB #177) | **1** | **1** | **3** | | **1** | **5** | **4** |  | **1** |  | **1** |  |  |  |  | **10** |
| **Losartan** (DB #678) | **3** | **2** | **6** | | **2** | **1** | **9** |  | **3** |  | **1** |  |  |  |  | **13** |
| **Candesartan** (DB #796) | **2** | **1** | **1** | | **2** |  | **2** |  |  | **5** |  |  |  | **2** |  | **2** |
| **Tasosartan** (DB #1349) | no information available | | | | | | | | | | | | | | | |
| **Celecoxib** (DB #482) | **6** | **2** | **6** | |  |  | **13** | **1** | **1** |  | **1** |  | **5** |  |  | **10** |
